# Stabilising large biologics through complimentary buffer component protection and rapid drying times

**DOI:** 10.1016/j.ijpx.2025.100374

**Published:** 2025-08-12

**Authors:** Laura Foley, Marina Steiner-Browne, Emmet O'Reilly

**Affiliations:** a SSPC the SFI Research Centre for Pharmaceuticals, Department of Chemical Sciences, Bernal Institute, University of Limerick, Limerick, Ireland; b Department of Chemical Sciences, Bernal Institute, University of Limerick, Limerick, Ireland

**Keywords:** Spray drying, Continuous manufacturing, Protein, Biopharmaceuticals, Stabilisation, Excipients, Fibrinogen

## Abstract

High processing temperatures restrict spray drying applications for heat sensitive biologics. This work highlights the potential of Phosphate Buffer Saline (PBS) as the sole stabilising excipient when spray drying large biologics via a complimentary buffer component effect. Fibrinogen (∼340 kDa) was spray dried in PBS at various temperatures and concentrations, followed by assessment of the protein's structural integrity by UV–Vis, ATR-FTIR, SEM, and PXRD analyses. Experimental findings demonstrate that fibrinogen can maintain structural integrity when spray dried at temperatures up to 60 °C (T_out_), when PBS is used as the sole stabilising excipient in combination with rapid drying rates. Results show a synergistic effect between the phosphate and salt components of the buffer when subjected to rapid drying rates mitigating protein aggregation and preserving protein secondary and tertiary structures. Stability studies conducted over 90 days indicated that powders stored under low humidity retained structural integrity. Findings provide valuable insights into the feasibility of spray drying large biologics through understanding the individual stabilising effects of PBS buffer components coupled with the rapid drying times. This approach offers a promising and scalable formulation strategy for the pharmaceutical industry in developing an alternative to freeze drying methods, offering advantages in cost, processing time, and product stability.

## Graphical abstract

**Figure:**
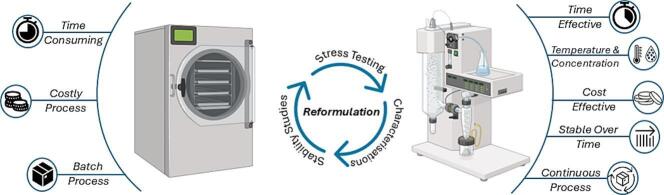

## 1 Introduction

Drying methods such as spray drying and freeze drying are employed when the stability of the liquid state is inadequate over time, storage requirements are costly and too stringent, or the product shelf life needs to be improved (Sharma et al., 2021; Duarte et al., 2025; Langford et al., 2018; Jadhav et al., 2024; Emami et al., 2023; Emami et al., 2018). They enable manufacturers to produce lifesaving medicines and therapies and distribute them worldwide to various climates and environments without the burden of cold storage, which is a common requirement when manufacturing biopharmaceuticals (Yu et al., 2021). Traditional cold chain storage approaches present challenges with respect to material handling, product quality over time and product spoilage affecting patient outcomes and increasing medicine costs (Pambudi et al., 2022; Hanson et al., 2017). As the number of biotherapeutics approved for use each year continues to grow, there is increased focus on the efficiency of drying methods.

Drying methods enable alternative delivery routes by converting liquid solutions to powders. For example, human plasma can be transformed into a powder “cake” allowing topical delivery and room temperature storage (Emami et al., 2023). While freeze drying remains the industry standard for drying most biologics, (Shukla, 2011; Bosch, 2014; Gamiz et al., 2019; De Jesus and Maciel Filho, 2014) its high costs, production volume limitations, poor process control and slow processing times have prompted exploration of alternatives (Brytan et al., 2025). In the field of biologics, spray drying has received less attention due to high processing temperatures that can cause denaturation and aggregation of heat sensitive biological materials.

Spray drying offers advantages as a one-step continuous manufacturing process (Parkes et al., 2022) compared to freeze drying. It exhibits a higher product throughput and can tailor the drying process to the desired product applications (De Jesus and Maciel Filho, 2014; Ledri et al., 2024; Ziaee et al., 2019). Spray drying has been used extensively in the production of small molecule pharmaceutical drugs however, high processing temperatures can lead to structural alterations in protein-based biopharmaceutical drugs resulting in reduced drug efficacy. The high cost, size and complexity of biologics, in tandem with low heat tolerances has limited the development of spray drying methodologies. Maintaining biological activity and structural integrity throughout manufacturing is essential for clinical functionality. Spray drying is approximately six times more cost effective per kilogram of water removal when compared to freeze drying (Barbosa et al., 2015). It produces a dry powder product that exhibits excellent long-term stability, can be stored and transported at ambient temperatures and, is structurally intact post processing.

Excipients play a crucial role in spray drying large biologics by preserving structural integrity and biological activity throughout the dehydration process and subsequent storage. Without the protective effect provided by these materials biologics would suffer denaturation and aggregation due to the thermal and mechanical stresses of spray drying. The most widely used excipients include disaccharides (trehalose, sucrose) which form protective glassy matrices through water replacement and vitrification mechanisms (Chang and Pikal, 2009; Vehring, 2008; Alhajj et al., 2021) surfactants (polysorbates) that prevent interfacial adsorption and denaturation (Lee et al., 2011), polymers (cyclodextrins, dextran) that provide thermal protections and reduce aggregation (Serno et al., 2011) and buffer systems (phosphate, citrate) that maintain optimal pH conditions. Other crucial excipients include amino acids especially during spray drying of biologics (Zhang et al., 2024; Ajmera and Scherließ, 2014). The strategic selection and combination of excipients enables the development of stable dry powder formulations with extended shelf-life, reduced cold chain requirements, and maintained therapeutic efficacy, making fragile biologics viable as commercial biopharmaceutical products.

PBS along with disaccharides and surfactants have been incorporated into spray dried formulations of biologics including monoclonal antibodies (Maury et al., 2005), vaccines (Sou et al., 2011) and enzymes (Rochelle and Lee, 2007). During the spray drying process biologics are exposed to rapid dehydration and high temperatures and shear forces. PBS and other excipients have been shown to mitigate against these effects through pH stabilisation, maintenance of ionic activity throughout the protein and excipient matrix formation, however the contribution from individual formulation components has not been studied in detail.

Fibrinogen is a glycoprotein that plays a critical role in haemostasis. As a biologic therapeutic, fibrinogen concentrates have become increasingly important in managing bleeding disorders including trauma induced coagulopathy and surgical haemorrhage. Fibrinogen is a large hexameric glycoprotein with a molecular weight of 340 kDa (Weisel and Litvinov, 2017). Three pairs of polypeptide chains make up fibrinogen, namely Aα, Bβ and γ linked by disulfide bridges. These form rod-shaped structures with globular structures on each end joined by coiled segments. Fibrinogen is a clotting factor that is triggered with an injury and prevents bleeding. Produced in the liver, it is an essential component of blood clotting. Fibrinogen is enzymatically converted to fibrin through thrombin mechanisms (Kattula et al., 2017; Pieters and Wolberg, 2019). Fibrinogen is not soluble in water but can be dissolved in warm saline solution and its solubility influenced by pH and temperature (Sigma-Aldrich, 2015). However, fibrinogen's complex structure, presents significant challenges for stability and formulation. Its sensitivity to temperature, pH fluctuations, and mechanical stress requires advanced processing techniques and stabilising excipients to preserve its biological activity. Advancing fibrinogen formulation technology is crucial for expanding its clinical applications and improving outcomes in the treatment of haemostatic conditions.

This study investigates the potential of PBS as the sole stabilising excipient in the spray drying of fibrinogen at various concentrations and processing temperatures. The contribution of the phosphate and salt components in tandem with rapid drying times are evaluated and compared to non-spray dried heat stressed samples. Conformational changes in the secondary and tertiary structure are evaluated by fluorescence spectroscopy (Dos Santos Rodrigues et al., 2023), UV–Vis spectroscopy (Kanjanawarut et al., 2013), in addition to Attenuated Total Reflectance Fourier Transform Infrared Spectroscopy (ATR-FTIR) (Kong and Yu, 2007). Dried particle morphology is assessed by Scanning Electron Microscopy (SEM) (Liu et al., 2010) and Powder X-Ray Diffraction (PXRD) is employed for assessment of product crystallinity (Liu et al., 2010).

## 2 Material and methods

### 2.1 Materials

Fibrinogen was supplied by Jansen Ireland. D- (+)-Trehalose dihydrate was purchased from Merk Life Sciences Ltd., Ireland. Phosphate Buffer Saline (PBS) tablets (pH 7.2–7.6), Magnesium Nitrate Hexahydrate and Glycine were purchased from Merk Life Science Ltd., Ireland. L-Arginine, high purity, low endotoxin and low metals, and Sucrose, high purity and low endotoxins derived from beet were purchased from Pfanstiehl. Ultra-pure type II Millipore water was obtained from a Milli-Q water purification system and used for the preparation of the PBS buffer. Spray drier feedstock solutions were filtered using 0.2 μm polyethersulfone (PES) membranes, Agilent Premium Syringe filters supplied by Captiva coupled with a Omnifix 10 mL syringes.

### 2.2 Methods

#### 2.2.1 Fluorescence Spectroscopy

Samples were analysed using an Agilent Technologies Cary Eclipse Fluorescence Spectroscopy. Stress tests samples were carried out at 25 °C, 45 °C, 50 °C, 55 °C and 60 °C. The fluorimeter was coupled with a Cary Temperature controller and a multi-cell holder was used to evaluate the unfolding/refolding transitions induced by the increase in temperature, specifically the tertiary structure of the protein. The samples were excited at 280 nm, and emission spectra were collected from 300 to 450 nm. Slit width of excitation and emission monochromators were set at 5 nm. The photomultiplier tube (PMT) was a set voltage of 600 nm.min^−1^. Measurements were caried out in triplicates, averaged, normalised by the maximum peak and smoothed using Savitzky-Golay. A temperature probe was used to ensure solution temperature accuracy, solution stabilisation was measured to be 5 min. All samples were dissolved in PBS buffer, to make a 1 mg.mL^−1^ solution, at room temperature and each spectrum took two minutes to collect. PBS buffers were prepared by dissolving one tablet in 200 mL of deionised water, this yields 0.01 M phosphate buffer, 0.0027 M potassium chloride and 0.137 M sodium chloride, pH 7.4, at 25 °C.

#### 2.2.2 UV–Vis spectrophotometry

Agilent Cary 60 UV–Vis spectrophotometry was used to evaluate the fibrinogen spectra and any baseline changes. All samples were dissolved in PBS buffer at room temperature (1 mg.mL^−1^). During stress testing samples were analysed at varying temperatures and aggregation was evaluated both visually and observing if there were changes in the spectrum baseline. Samples post spray drying at varying temperatures and concentrations were evaluated in triplicate, averaged, normalised (by maximum peak) and plotted against fibrinogen as-received (AR) sample. The sample was prepared by adding fibrinogen (1 mg.mL^−1^) as received to PBS buffer, this sample was neither stressed nor spray dried. Spectra were collected from 200 to 600 nm and the scan rate was 24,000 nm.min^−1^.

#### 2.2.3 Spray drying

Liquid feed stock solutions used for these tests were heated to 37 °C using a water bath. Once the solution temperature reached 37 °C, fibrinogen was added to the solution and stirred for one hour at 150 rpm. The solution was then filtered using a 0.2 μm polyether sulfone (PES) membrane filter before spray drying. A sample of fibrinogen dissolved in PBS buffer, ultra-pure water, phosphate solution and salt solution (potassium chloride and sodium chloride) were prepared and are outlined in Table 1. The Buchi B-290 mini spray dryer was used in open loop mode. This was used in conjunction with a dehumidifier (−4 °C), which condensed water vapour in the air stream with absolute water content ranging between 25 and 27 %. A 2-fluid nozzle with a 0.7 mm nozzle tip diameter was used throughout the study.

**Table 1:** Outline of all variable spray drying processing parameters.

Run ID	Inlet Temperature T_out_ °C (± 1 °C)	Outlet Temperature T_out_ °C (± 1 °C)	Protein Content *w*/*v* %	Solvent
	***Temperature Investigation***			
FIBSD45	72	45	0.2	PBS Buffer
FIBSD50	81	50	0.2	PBS Buffer
FIBSD60	94	60	0.2	PBS Buffer
FIBSD70	112	70	0.2	PBS Buffer
FIBSD80	129	80	0.2	PBS Buffer

	***Protein Concentration Investigation***			
FIBSD0.2	76	50	0.2	PBS Buffer
FIBSD0.5	75	50	0.5	PBS Buffer
FIBSD1.0	78	50	1.0	PBS Buffer
FIBSD2.0	79	50	2.0	PBS Buffer

	***Spray Drying in Water***			
FIBSDW0.2	76	50	0.2	Ultra-Pure Water
FIBSDW2.0	78	50	2.0	Ultra-Pure Water

	***Spray Drying in Phosphate/ Salts***			
FIBSDPhos	78	50	0.2	Phosphate solution
FIBSDSalts	78	50	0.2	Potassium chloride and sodium chloride solution

Atomisation gas flow rate was kept constant at 473 L.h^−1^ with a feed rate of 1.5 mL.min^−1^ and the aspirator set to 100 % (35.0 m^3^.h^−1^ or 35,000 L.h^−1^). A range of temperatures and concentrations were investigated, and the variable operating parameters are outlined in Table 1 below. The spray drying system was first stabilised using the ultrapure water and once stable the feed stock was then pumped to the drying chamber. All samples were collected after 20 to 25 min of spray drying.

#### 2.2.4 Scanning Electron Microscopy (SEM)

The morphological structure of as-received and spray dried fibrinogen samples was examined using SEM, Hitachi SU-70; a high-resolution field emission electron microscope. Operating voltage was set at 5 kV with a 10 mm working distance. Samples were gold‑palladium coated prior to analysis to avoid any overcharging that may occur.

#### 2.2.5 Powder X-Ray Diffraction (PXRD)

Structural powder determination was characterised using an Empyrean diffractometer (PANalytical, Phillips). The diffractometer was used at room temperature in reflection mode with Cu Kα radiation (λ = 1.5406 Å) in 2θ range of 5° to 45°. The tube voltage and current were set to 45 kV and 40 mA, respectively. The step size was 0.03° with a scan speed of 0.05° s^−1^. Flat layer of powders was prepared by gently pressing them on a silicon zero-background disc using a clean glass slide.

#### 2.2.6 Stability Testing

The Amebis Temperature Controlled Cabinet U063 was used for this study as a stability chamber to investigate the stability of the spray dried samples over time. ICH guidelines for accelerated stability testing were followed. The chamber was set at 40 °C (tolerance of ±2 °C), while the relative humidity was set at 75 % (tolerance ±5 %) and was maintained using saturated sodium chloride solutions (Ajmera and Scherließ, 2014). Large sample jars with sealed lids were used to store the samples along with the saturated solutions of sodium chloride. Prior to stability studies, jars were placed in the stability chamber for 24 h to ensure the environment stabilised beforehand. Stability testing was carried out for 90 days, with samples taken on Day 0, Day 7, and Day 90.

#### 2.2.7 Attenuated Total Reflectance Fourier Transform infrared Spectroscopy (ATR-FTIR)

The ATR-FTIR spectra were obtained using a Nicolet iS50 FTIR spectrometer (ThermoFisher Scientific) with OMNIC software. This was used to determine changes in the protein's secondary structure caused by structural denaturation/degradation during the spray drying process. Spectra were collected in 64 scan cycles and were averaged and plotted. 2-propanol (IPA) was used to clean the crystal before and after sample analysis. A background was collected and subtracted from each run.

## 3 Results and discussion

### 3.1 Preliminary screening studies

#### 3.1.1 Fibrinogen stress testing

Initial temperature screening is critical to inform the temperature at which the protein degrades and thereby inform critical processing parameters such as the spray drying outlet temperature (T_out_). Fibrinogen has previously been reported to degrade between 45 °C and 55 °C (Marx et al., 2008). To identify the temperature at which the onset of denaturation occurs, stress tests were performed on heated samples which were then characterised using fluorescence and UV–Vis spectroscopy. Denaturation in fibrinogen can be identified by a shift in the maximum fluorescence (λ_max_) corresponding to denaturation of the secondary and tertiary structures (Wu et al., 2019).

**Fig. 1:**
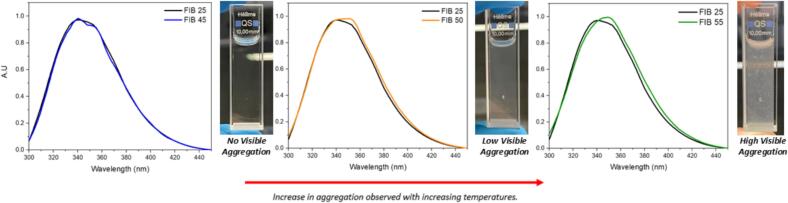
Stress testing of fibrinogen dissolved in PBS buffer by fluorescence spectroscopy. Fibrinogen was heated from 25 °C to 55 °C and the emission spectra were plotted. Captures of cuvettes during the fibrinogen stress tests across increasing temperatures accompany these plots.

All samples were heated from room temperature to a desired investigation temperature up to 55 °C. Samples remained at this temperature for 5 min prior to analysis to stabilise the solution. Fig. 1 shows a shift in the fluorescence λ_max_ peak from 340 to 349 nm at 50 °C due to the onset of denaturation of the protein tertiary structure. This is further observed at 55 °C. Aggregation resulting from denaturation can also be identified visually through increased aggregation as temperature increases (Fig. 1).

#### 3.1.2 Excipient screening

To overcome temperature-induced denaturation issues associated with higher drying temperatures, the protective effect of various excipients was investigated based on previous literature reports (Kenmi Miyano et al., 1992; Foley et al., 2024; Sharma et al., 2024; Chen et al., 2021). Higher drying temperatures will produce a lower moisture content and increase product stability and shelf life. For effective spray drying of biologics, a minimum T_out_ temperature of 50 °C is required for aqueous solutions to facilitate lower moisture content and enhance subsequent product shelf life. Trehalose, sucrose and amino acids glycine and L-arginine were added to fibrinogen dissolved in PBS. Ratios investigated are based on literature and previous experimental findings. Trehalose is a commonly used excipient with a high glass transition temperature that has previously been used to spray dry large molecules was investigated at a 1:2 ratio *w*/w (fibrinogen: trehalose) (Foley et al., 2024). Results showed denaturation was still occurring at ∼50 °C (Fig. S1 (A)). Similarly, for the monosaccharide sucrose, another established excipient, denaturation was observed at ∼50 °C with the same 1:2 *w*/w ratio (Fig. S1 (B)). The amino acids, glycine and L-arginine, that have previously been reported to stabilise fibrinogen in solution (Zhang et al., 2024; Kenmi Miyano et al., 1992; Peng and Beckett, 2021) glycine was investigated both individually at 1:0.5 w/w (protein: amino acid) and combined with L-arginine at 1:0.5:0.5 (protein: amino acid: amino acid). Results show protein denaturation occurred before 50 °C for both studies (Fig. S2). Further investigation into literature studies used to stabilise fibrinogen resulted in a study that examined a process for heat treating fibrinogen up to 90 °C in solution (Kenmi Miyano et al., 1992). This was experimentally tested and although fibrinogen could withstand higher temperatures with no effect to the proteins structure, this formulation was not suitable for spray drying producing an aerated/moist sugar fibres product despite the promising initial results (Fig. S3).

**Fig. 2:**
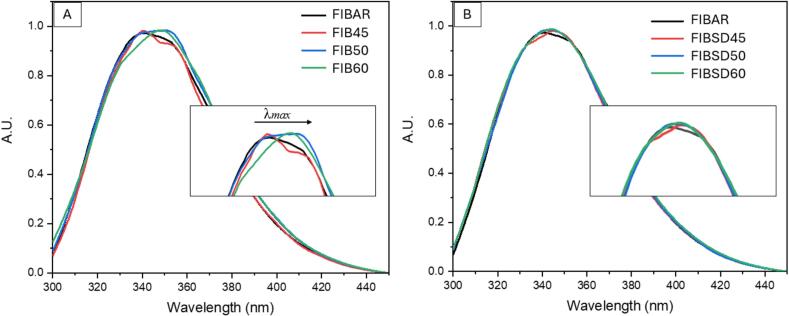
Fluorescence spectroscopy of fibrinogen dissolved in PBS (A) Stress test and (B) post spray drying at various temperatures.

Pharmaceutical formulation strategies support the limited use of excipients in the production of therapeutic dosages, as in some oral dosage forms interactions between excipients and the active drug ingredient can affect its bioavailability, adsorption, dissolution and toxicity (Kalasz and Antal, 2006; Abrantes et al., 2016). Although PBS did not stabilise fibrinogen during solution stress tests, buffering agents and salts have been reported to stabilise biomolecules during the spray drying process (Pinto et al., 2021). Fibrinogen dissolved in PBS buffer was spray dried at varying temperatures between 45 and 60 °C without the addition of further excipients. A solution of 0.2 % *w*/*v* of fibrinogen was dissolved in PBS buffer at 37 °C and stirred for one hour, filtered, and spray dried. The final dry powder product was redissolved in PBS buffer at 1 mg.mL^−1^ and compared to fibrinogen solutions examined during the stress tests. Fig. 2 shows a comparison of fibrinogen in PBS subjected to elevated temperatures during stress testing compared to the spray dried samples using PBS as the sole excipient. Results show that unlike the stress testing studies, there was no protein degradation in spray dried samples at operating temperatures up to 60 °C. A possible reason for this may lie in the spray drying process itself.

Previous studies have shown various buffering agents acting as excipients during the spray drying process (Chaudhari and Patil, 2012). Spray drying rapidly dries a liquid solution into a solid using hot gas. This process occurs significantly faster when compared to the stress test. A study conducted by Mutukuri et al. compared the use of buffers as excipients in spray drying versus freeze drying and noted higher levels of protection during the spray drying process due to its ability to rapidly dry droplets, rather than prolonged freezing and sublimination that occurs during freeze drying. This prolonged exposure time has a negative effect on the stability of the protein in the solid form (Mutukuri et al., 2023). A review conducted by Ohtake et al. also stated that it is highly plausible that the protein and the buffer salt form a hydrogen bond thus substituting for the loss of water molecules indicating structural stabilisation (Izutsu et al., 2009; Ohtake et al., 2011). These theories are discussed further in section 3.4.

#### 3.1.3 The effect of Time and Temperature

**Fig. 3:**
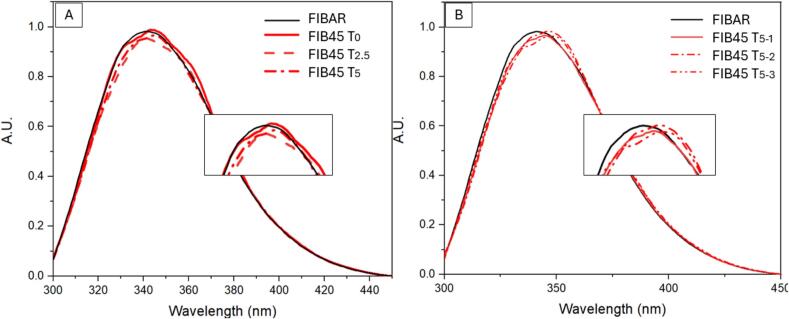
(A) fluorescence spectroscopy plot of fibrinogen dissolved in PBS buffer examined at 45 °C over a five-minute interval, (T_0_ = 0 min, T_2.5_ = 2.5 min and T_5_ = 5 min) and (B) fluorescence spectroscopy triplicate plotted after five minutes at 45 °C (triplicates are represented by 45 T_5__–__1_, 45 T_5__–__2_, and 45 T_5__–__3_).

Fig. 2 demonstrates that the time Fibrinogen is exposed to high temperatures plays a significant role in observed denaturation. To further investigate this observation, the effect of temperature on non-spray dried, heat stressed fibrinogen within a five-minute period allocated for solution stabilisation was investigated. Three spectra were taken at 0, 2.5 and 5 min, once the desired temperature was reached. Replicates after five minutes at 45 °C (45 T_5__–__1_, 45 T_5__–__2_ and 45 T_5__–__3_) were also collected and plotted separately. Results are represented in Fig. 3 below.

In Fig. 3 (A) a shift in λ_max_ is occurring as time progresses, overall showing the effect of temperature on fibrinogen denaturation over time. In Fig. 3 (B) the replicates plotted do not align, and minor λ_max_ shifts after the five-minute stabilisation period are observed. Results from this study provided information of fibrinogen denaturation patterns over time when compared to the spray drying process. Spray drying is a rapid drying process therefore fibrinogen may only experience high temperatures for short periods of time. The residence time of the drying air within the Buchi B-290 spray chamber is approximately 1.5 s (Dr Cordin Arpagaus, 2008). This is an indication on why the stress test studies are not comparable to the results obtained for spray drying.

### 3.2 Effect of Temperature and Concentration on Spray Dried Fibrinogen

#### 3.2.1 Effect of Temperature on Spray Drying Fibrinogen

**Fig. 4:**
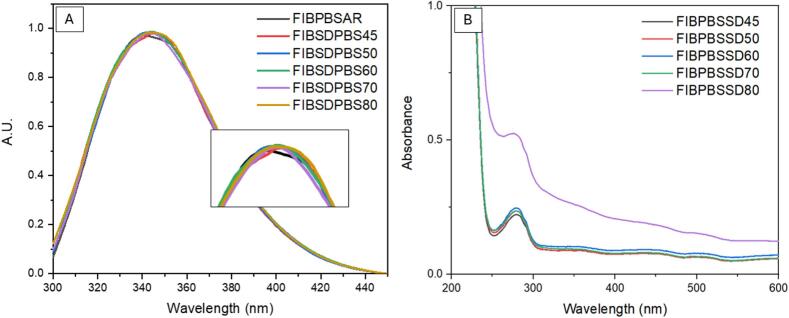
(A) Fluorescence spectroscopy of spray dried fibrinogen in PBS buffer at varying temperatures and (B) UV–Vis spectrum of spray dried fibrinogen in PBS buffer at varying temperatures.

Fibrinogen in PBS was spray dried at five different temperatures (45, 50, 60, 70 and 80 °C) to further assess the protection capabilities of PBS buffer. Spray dried samples were characterised by fluorescence spectroscopy and UV–Vis spectrophotometry (Fig. 4). Additional analysis including ATR-FTIR, SEM and PXRD was used to further assess fibrinogen secondary structure, morphology and crystallinity post processing. The fluorescence spectroscopy data across all five temperatures was compared to fibrinogen as-received (AR). Fig. 4 (A) shows a shift in λ_max_ from 340 to 349 nm is beginning to occur at 70 °C and is also observed at 80 °C. As previously outlined this is indicative of the onset of denaturation. Fig. 4 (B) shows the UV–Vis spectra where a significant change in the baseline and increase in absorption is occurring at 80 °C. The change in absorption relates to the aggregation of fibrinogen due to protein degradation. This is further supported by ATR-FTIR data examining the effects of temperature on the secondary structure of fibrinogen at 70 °C.

**Fig. 5:**
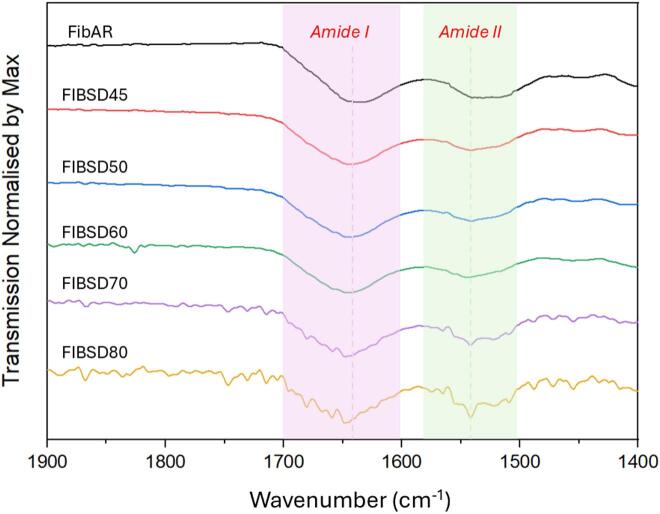
FTIR data of spray dried fibrinogen at varying temperatures plotted against fibrinogen as received (AR) and fibrinogen in PBS buffered salts spray dried (SD) at various temperatures.

Fig. 5 shows the corresponding FTIR spectra of the spray dried fibrinogen at the five processing temperatures investigated in addition to fibrinogen (AR). The protein's amide I and amide II bands are located at 1650 cm^−1^ and 1540 cm^−1^ respectively. In the amide I region, 1600–1700 cm^−1^, a shift in maximum peak is seen with an increase in temperature. Notably, at 80 °C a shift is observed showing a disruption to the secondary structure of fibrinogen. Specifically at ∼1650 cm^−1^ which is specific to α-helix region of the fibrinogen secondary structure (Litvinov et al., 2012).

**Fig. 6:**
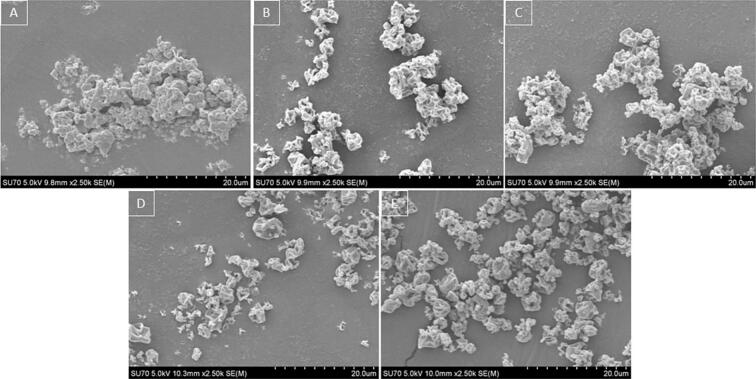
SEM images of spray dried fibrinogen dissolved in PBS buffer (A) SD at 45 °C, (B) SD at 50 °C, (C) SD at 60 °C, (D) SD at 70 °C and (E) SD at 80 °C.

Fig. 6 shows the morphology of the spray dried fibrinogen particles at the five different temperatures investigated. All spray dried particles exhibited a crumpled morphology irrespective of processing temperature. This morphology can be related to rapid particle drying of the salts within the PBS buffer solution and low fibrinogen concentration. With an increase in temperature, a decrease in particle cohesiveness is observed. At higher temperatures, less product is sticking to the drying chamber and cyclone walls, along with less particle agglomeration. Sample uniformity and size also became more consistent with an increase in temperature.

The crystallinity of the spray dried powder was examined using PXRD and diffractograms of each temperature are plotted and compared in Fig. S4, Supplementary Information. The crystallinity of the final dry powder product is a combination of amorphous fibrinogen and crystalline PBS buffer salts for all samples. Stability study results are reported in section 3.3.

#### 3.2.2 Effect of Concentration on Spray Drying Fibrinogen

Spray drying fibrinogen at varying concentrations was performed to assess the effect of protein solid content on the final structural integrity. Although the temperature studies showed promising results when dried in PBS, investigations into spray drying fibrinogen at higher concentrations is beneficial for assessing industrial feasibility. Increasing protein concentrations and thereby reducing production times and costs, underlies the industrial viability of many spray drying operations.

**Fig. 7:**
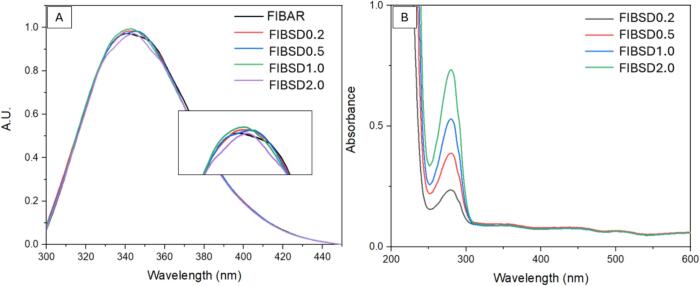
Spray dried fibrinogen in PBS buffer at varying concentrations (A) fluorescence spectroscopy and (B) UV–Vis spectrum.

Four concentrations were examined, 0.2 % *w*/*v*, 0.5 % w/v, 1 % w/v and 2 % w/v. Fig. 7 shows the fluorescence spectroscopy and UV–Vis spectra for each concentration. Results show in Fig. 7 (A) that no shift in the maximum (λ_max_) peak is observed. Each concentration was spray dried at an outlet temperature of 50 °C. No change in the baseline is observed in Fig. 7 (B), indicating no increase in aggregation occurs with an increase in concentration. The observed increase in the maximum peak intensity in Fig. 7 (B) is proportional to the increasing fibrinogen concentration.

**Fig. 8:**
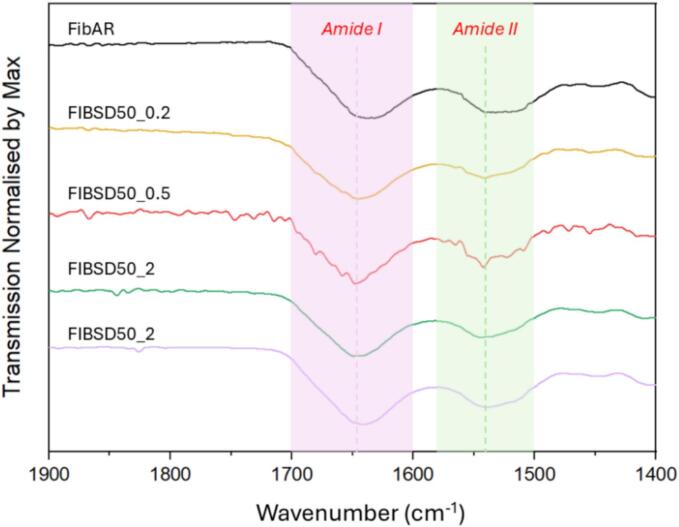
FTIR data of spray dried fibrinogen at varying concentrations plotted against fibrinogen as received.

Fig. 8 shows FTIR spectra for each concentration in which little variation in the amide I (1600–1700 cm^−1^) region was observed which correlates to the fluorescence spectroscopy and UV–Vis data reported above. Therefore, it was observed that there was no significant effect of concentration on the structural integrity of fibrinogen spray dried at 50 °C. FTIR spectra include a comparison of the spray dried samples at varying concentrations to the fibrinogen AR and the PBS buffered salts.

**Fig. 9:**
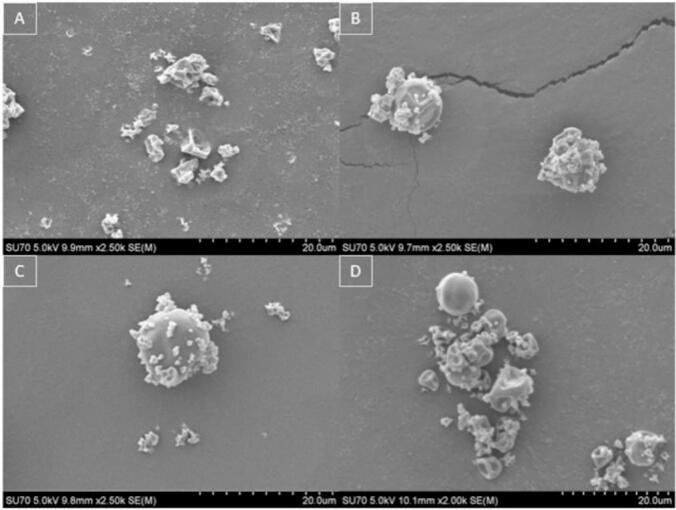
SEM images of spray dried fibrinogen in PBS solution at varying concentrations (A) 0.2 % *w*/*v*, (B) 0.5 % w/v, (C) 1 % w/v and (D) 2 % w/v.

Fig. 9 shows SEM images of the particle morphology of the final dry powder product collected. A change in the particle morphology is observed with an increase in the solid content of fibrinogen. In Fig. 9 (A) small, shrivelled particles are observed similar to SEM images obtained in Fig. 6**.** This is due to the lower overall solid content of the solution, with faster droplet drying and rapid particle formation. However, moving from (A) to (B), (C) and (D) in Fig. 9 an increase in smooth spherical particles is observed. These larger smoother particles are increased due to the presence of higher fibrinogen concentrations, thus influencing the particle morphology and size. A study conducted by Vicente et al. reported on the effects of feed concentration on particle morphology which aligns with our findings that particle shrivelling occurred at lower solid concentrations (Vicente et al., 2013). The particle morphology has also been discussed presenting a more shrivelled particle due to thin particle shells at lower concentrations and more round spherical particles at higher overall solute concentrations.

In terms of the final powder crystallinity, the powder remains semi-crystalline with the amorphous fibrinogen and crystalline PBS buffer salts post processing. Fig. S5, Supplementary Information, depicts diffractograms for the four varying concentrations of spray dried fibrinogen. This work shows the capability of spray drying fibrinogen at a 0.2 % w/v up to a 2 % w/v with no changes to the structural integrity of the protein, crystallinity and minor adjustments to the particle morphology based on solid content. Overall, these results show that fibrinogen can be spray dried at an outlet processing temperature up to 60 °C and protein solid content up to 2 % w/v while maintaining the structural integrity of the molecule in the presence of PBS. Stability studies for concentration were conducted and presented in section 3.3 below.

### 3.3 Stability Studies

**Fig. 10:**
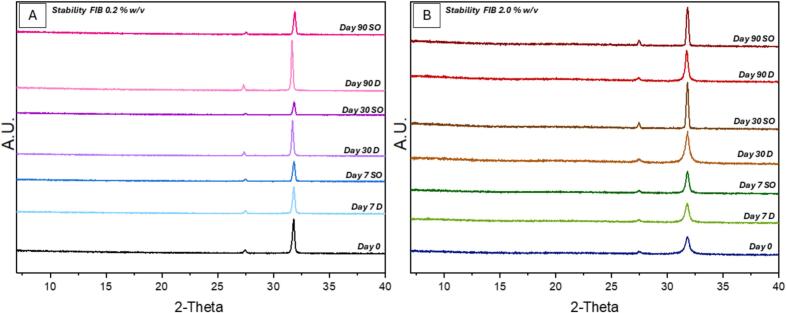
PXRD diffractograms of the stability of spray dried fibrinogen over time at different concentrations. (A) fibrinogen at 0.2 % *w*/*v* stability and (B) fibrinogen at 2 % w/v stability, Day 0, Day 7, Day 30 and Day 90 are compared in the desiccator (D) and stability oven (SO).

Diffractogram results show a comparison of the stability of fibrinogen over time in the stability oven (Fig. 10 (B)) at elevated temperatures (40 °C) and humidity (75 %) against the stability of the spray dried powders stored in the desiccator overtime (Fig. 10 (A)). The stability was examined on Day 0, Day 7, Day 30 and Day 90. This data indicates the feasibility of long-term storage and shipping of the spray dried powders similar to the current freeze dried fibrinogen samples. The stability was also examined via fluorescence spectroscopy where spectra was collected for samples stored in the desiccator after spray drying (Day 0), Day 30 and Day 90. No shift in the maximum peak (λ_max_) was observed (Fig. S6, Supplementary Information). Samples stored in the stability oven were also analysed on the fluorescence spectroscopy however due to limited amount of powder available, analysis was carried out on Day 0 and Day 90. There was a slight shift in the λ_max_ peak in the samples stored in the stability oven, showing a disruption to the protein's structure at elevated temperatures and humidity after 90 days, although no change is observed in the diffractograms in Fig. 10 (B) below.

As previously mentioned, there are many advantages to spray drying over freeze drying in terms of cost and time efficiency therefore, this work advances the ability to be able to produce a stable, dry powder fibrinogen product suitable for applications in patches like those manufactured by Johnson & Johnson and Corza Medical. No change was seen over the stability studies in terms of crystallinity. Fluorescence spectra were collected after 30 and/or 90 days showed no shift in the λ_max_ peak were observed for the samples stored in the desiccator; indicating that the protein was stable.

### 3.4 Stabilising Effects of PBS on Spray Dried Fibrinogen

The PBS is crucial for preserving fibrinogen's structural integrity during spray drying by acting as an excipient. Results show that fibrinogen cannot be spray dried in water alone without the addition of PBS (Fig. S9). Buffers primarily stabilise proteins in solution by maintaining their pH (Mutukuri et al., 2023; Pavani et al., 2021; Zbacnik et al., 2017) however, several mechanisms have been proposed to explain how they specifically protect a protein during the drying process. These include stabilising the proteins surface charge, and minimising aggregation caused by thermal and shear forces (Ugwu and Apte, 2004). However, results in this study show that droplet drying and particle formation also play a crucial role in protecting fibrinogen during the spray drying process. Buffer salts are highly soluble and evaporate rapidly causing an outer shell to form in the early stages of drying. The outer shell will be mainly composed of the buffer salt due to this rapid drying, while the protein remains in the inner core drying slower from the crust inwards (Vehring et al., 2007; Huang, 2011). Other theories suggest that buffers may be stabilising the protein through hydrogen bonding (Jorgensen et al., 2009). As water is lost during the drying process, PBS has the potential to form new bonds with the protein replacing those lost as the water dries (Ohtake et al., 2011).

### 3.5 Spray Drying Fibrinogen in Phosphate and Fibrinogen in Salt

**Fig. 11:**
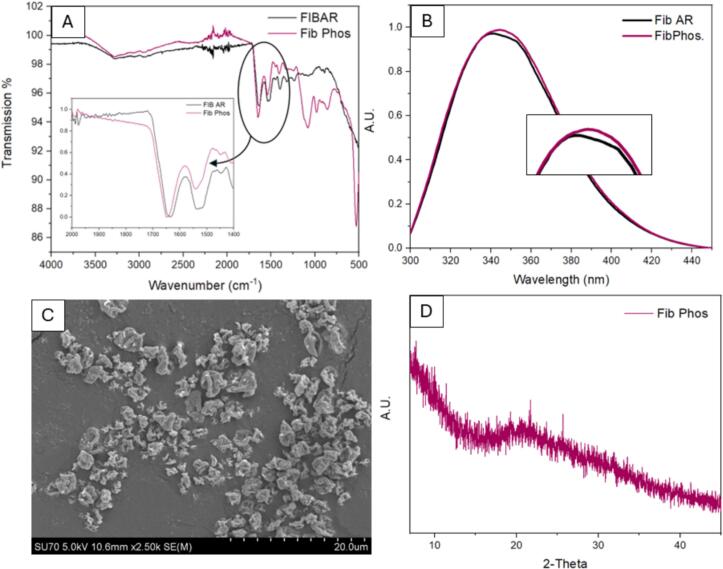
Results of spray dried fibrinogen in phosphate (0.01 M) at 0.2 % *w*/*v*. (A) ATR-FTIR spectrum comparison of spray dried fibrinogen in phosphate vs fibrinogen as received, (B) fluorescence spectroscopy plot, (C) SEM image of spray dried fibrinogen in phosphate and (D) PXRD diffractograms of spray dried fibrinogen in phosphate.

To identify which components of PBS enable processing at higher temperatures, fibrinogen (0.2 % *w*/*v*) was spray dried in both phosphate and salt solutions while maintaining a T_out_ of 50 °C. Spray dried samples were examined for secondary and tertiary denaturation as previously outlined. Fig. 11 shows the results of spray drying fibrinogen in phosphate solution alone. In Fig. 11 (A) FTIR data identifies a shift due to potential unfolding or a disruption in the fibrinogen's secondary structure induced by temperature. However, the fluorescence spectra show the tertiary structure of the fibrinogen was not degraded as no shift in the maximum peak (λ_max_) is observed in Fig. 11 (B) however, disruptions in the secondary structure would mean disruptions to the tertiary structure also. SEM images show dimpled morphology previously observed for other samples (Fig. 6) at 0.2 % w/v concentration. PXRD in Fig. 11 (D) shows an amorphous fibrinogen without the presence of salt.

**Fig. 12:**
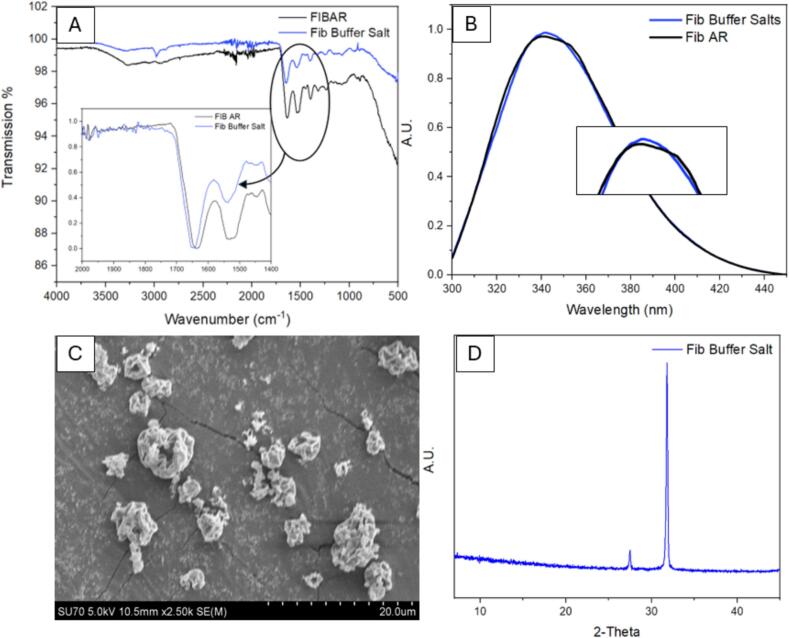
Results of spray dried fibrinogen in salt (0.0027 M of potassium chloride and 0.137 M of sodium chloride) at 0.2 % w/v. (A) ATR-FTIR spectrum comparison of spray dried fibrinogen in salt vs fibrinogen as received, (B) fluorescence spectroscopy plot, (C) SEM image of spray dried fibrinogen in phosphate and (D) PXRD diffractograms of spray dried fibrinogen in phosphate.

Fig. 12 shows the results of spray drying fibrinogen in PBS component salts sodium chloride and potassium chloride without the presence of phosphate. Fig. 12 (A) shows the FTIR analysis indicating a shift in the secondary structure of the protein similar to that observed in Fig. 11 (A). The fluorescence spectra in Fig. 12 (B) shows that the overall tertiary structure of the fibrinogen is intact with no shift in the maximum peak (λ_max_) or degradation observed, similarly as mentioned above this may not be the case and disruptions to the secondary structure could be assumed to als be occurring. SEM images show that the dimpled morphology which has been present in all spray dried samples. PXRD diffractograms are semi-crystalline owing to crystalline salts and an amorphous fibrinogen.

A shift in the λ_max_ peak fluorescence spectra (Fig. 12 (B)) is not observed implying that a level of protection is offered by each component of the buffer individually. However, FTIR results show a shift in the secondary structure of the protein in both formulations (salt and phosphate) which was not observed when fibrinogen was spray dried in PBS buffer. This allows us to conclude that both the phosphate and salts combined are crucial to maintaining the structural integrity of fibrinogen during the spray drying process. In both cases, spray drying in either phosphate or salt, unfolding is occurring with the applied temperature causing disruption to the secondary structure of the protein but refolding to its previous tertiary structure (Stirnemann et al., 2014). The addition of the phosphate buffer provides sufficient protection for refolding of the secondary structure or prevents irreversible unfolding from occurring, and no shift in the FTIR spectra is seen up to 60 °C post spray drying (Fig. 5). Although no overall degradation is observed for the protein post processing for either salt or phosphate, the observed change in secondary structure can have severe effects in terms of drug efficacy and compromise patient safety.

The replacement of hydrogen bonds lost during the drying process through excipient bonding is regarded as the most likely mechanism preventing secondary denaturation in proteins (Ohtake et al., 2011). However, if this theory was broadly applicable, phosphate alone should be sufficient to stabilise the protein. Results show that a synergistic protective effect is occurring with phosphate components likely replacing the water-protein hydrogen bonds lost during the drying process. Concurrently, sodium and potassium chloride salts contribute to structural stability through surface charge effects and altered drying kinetics. During rapid drying, the highly soluble salts migrate to the droplet surface creating a protective layer and limiting thermal stress upon the protein. The complimentary effects of the phosphate and salt components in addition to rapid drying and low residence time heat exposure, permit effective drying of fibrinogen at higher temperatures while maintaining structural integrity.

## 4 Conclusion

This study demonstrates the complimentary buffer component protection of PBS when spray drying fibrinogen. Neither phosphate nor salt components alone provide the same level of protection as complete PBS when combined with rapid drying times. This enabled spray drying of fibrinogen at outlet temperatures up to 60 °C without compromising secondary or tertiary structures. A comparison of stress tested fibrinogen and spray dried samples, demonstrates that rapid drying times in tandem with the complimentary buffer component effect of PBS are a requirement for maintaining the proteins stability. The spray dried fibrinogen powders remained stable over a 90-day period, with no significant denaturation observed, confirming the long-term storage feasibility of the product. These findings collectively demonstrate that spray drying with PBS buffer offers a viable alternative to freeze drying for producing stable, dry fibrinogen powders, with potential advantages in cost, processing time, and elimination of cold chain requirements for biopharmaceuticals. The findings from this study not only advance the understanding of fibrinogen's behaviour under spray drying conditions but also highlight the critical role of buffers in preserving protein integrity during processing. This work paves the way for more efficient production methods for fibrinogen-based products in the pharmaceutical and medical industries.

## CRediT authorship contribution statement

**Laura Foley:** Writing – review & editing, Writing – original draft, Visualization, Validation, Methodology, Investigation, Data curation, Conceptualization. **Marina Steiner-Browne:** Writing – review & editing, Methodology, Conceptualization. **Emmet O'Reilly:** Writing – review & editing, Supervision, Funding acquisition, Conceptualization.

## Declaration of competing interest

The authors declare that they have no known competing financial interests or personal relationships that could have appeared to influence the work reported in this paper.

## Data Availability

Data will be made available on request.

## Appendix A Supplementary data

Stabilising large biologics through complimentary buffer component protection and rapid drying times Image 1
