# Supplementary material for: Stabilising large biologics through complimentary buffer component protection and rapid drying times
Source: Int J Pharm X. 2025 Aug 12;10:100374. doi: 10.1016/j.ijpx.2025.100374 (PMC12392680; doi:10.1016/j.ijpx.2025.100374)
Supplement: Supplementary file 1 — Stabilising large biologics through complimentary buffer component protection and rapid drying times [file mmc1.docx]

Supplementary information

**Stabilising Large Biologics through Complimentary Buffer Component Protection and Rapid Drying Times**

*Authors:*

Laura Foley^1^, Marina Steiner-Browne^2^ and Emmet O’Reilly^1^.

^1^SSPC the SFI research centre for Pharmaceuticals, Department of Chemical Sciences,

Bernal Institute, University of Limerick, Limerick, Ireland.

^2^Department of Chemical Sciences, Bernal Institute, University of Limerick, Limerick, Ireland.

Table of Contents

[Fibrinogen Excipient Screening](#_Toc195181012)

[Heat Treating Fibrinogen](#_Toc195181013)

[Powder X-Ray Diffraction (PXRD)](#_Toc195181014)

[Temperature Variation Results](#_Toc195181015)

[Concentration Variation Results](#_Toc195181016)

[Fluorescence spectrometry](#_Toc195181017)

[Stability Oven: Spray dried fibrinogen spectra](#_Toc195181018)

[Desiccator: Spray dried fibrinogen spectra](#_Toc195181019)

[Scanning Electron Microscope (SEM)](#_Toc195181020)

[Spray Drying Fibrinogen in Water](#_Toc195181021)

# Fibrinogen Excipient Screening


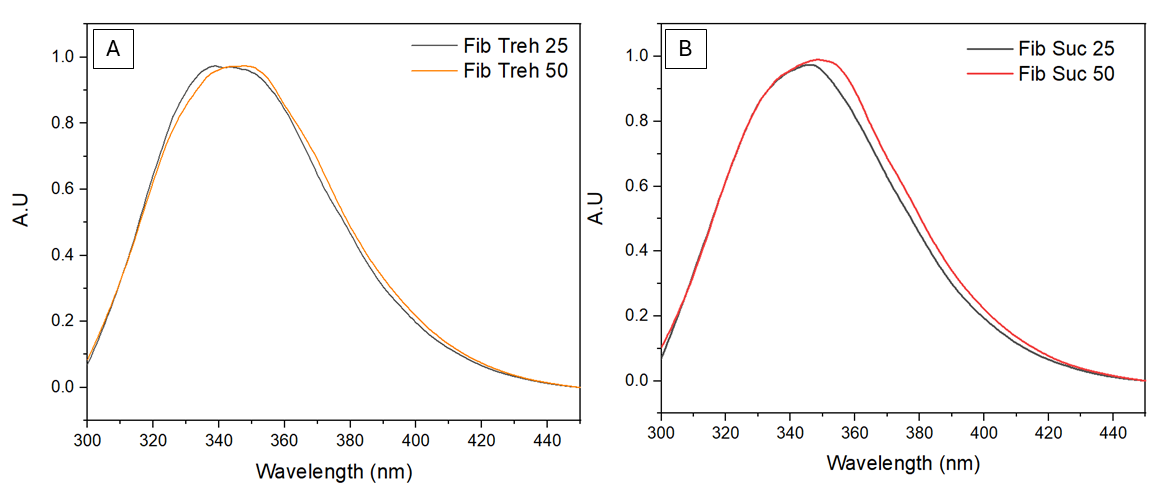


*Figure S1, (A) Spectra of stress tested fibrinogen with trehalose at 25 and 50 °C and (B) Spectra of stress tested fibrinogen with sucrose at 25 and 50 °C.*


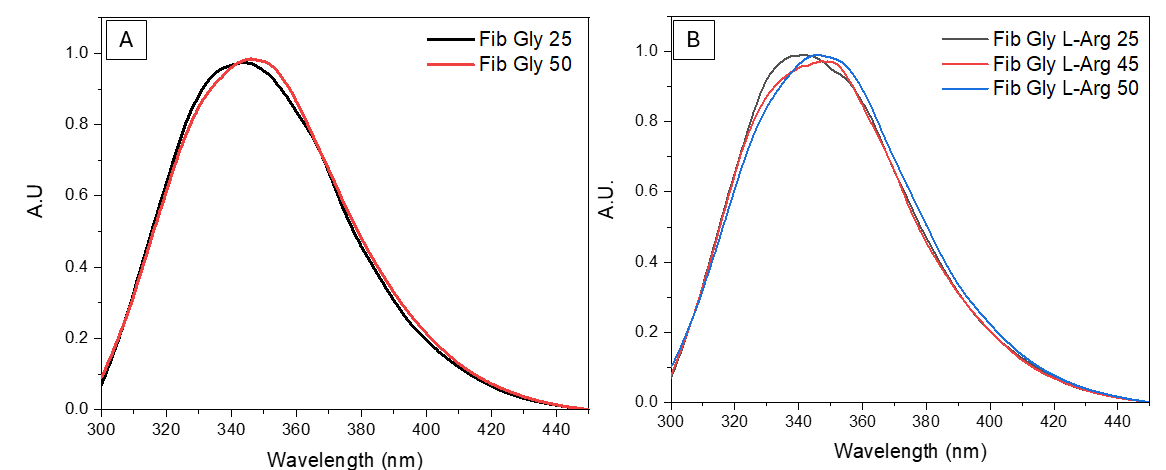


*Figure S2 (A) Spectra of stress tested fibrinogen with glycine at 25 and 50 °C and (B) Spectra of stress tested fibrinogen with glycine and L-arginine at 25 and 60 °C.*

## Heat Treating Fibrinogen

Further investigation into studies used to stabilise fibrinogen resulted in a study that examined a process for heat treating fibrinogen [1]. These results informed our solution formulation as follows: fibrinogen 1 mg.mL^-1^, sucrose 2.4 mg.mL^-1^, glycine 0.4 mg.mL^-1^ and lastly magnesium nitrate 100 mM; the solution pH was 6.9. This method was used for viral inactivation (VIN) studies however, this formulation could possibly provide a suitable formulation for spray drying fibrinogen. Therefore, following the study conducted by Miyano *et al.* and the formulation of protein to excipients combinations, we confirmed we could prevent protein degradation in the liquid form. In Figure S1 (A) no peak shift was observed up to 50 °C (blue). However, at 60 °C the maximum peak was shifted (green); therefore, we can assume structural integrity of the protein is compromised at this temperature. These results were used to inform our spray drying T_out_ processing parameter, where temperatures up to 50 °C should not cause disruption to the fibrinogen. No visible aggregation was observed in the cuvette at 60 °C although a shift in maximum peak was observed (Figure S1 (B)).


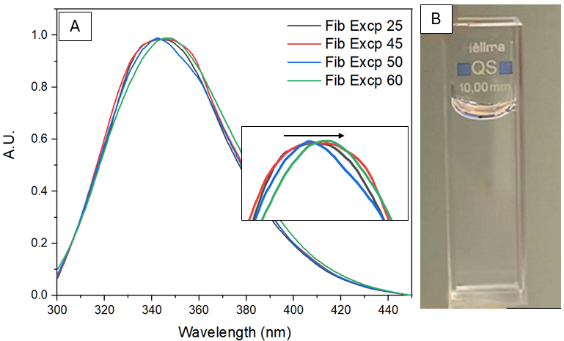


*Figure S3, (A) Spectra of stress tested fibrinogen from 25 to 60 °C. Formulation consisted of fibrinogen at 0.2% w/v with the addition of salt: magnesium nitrate (100 Mm), sugar: sucrose (60% w/v) and amino acids: glycine (10% w/v). (B) Quartz cuvette colourless after being exposed at 60 °C.*

Although the formulation was suitable from preventing heat denaturation at our desired temperature range, our investigation into spray drying this combination of protein to excipients was not suitable for the spray drying process. Spray drying this formulation produced a solid matrix rather than a powder. This could be due to the sucrose’s low glass transition (T_g_) temperature, causing the sucrose to turn into a glassy, sticky state that solidified once cooled down into room temperature. Therefore, giving an insight into some of the limitations of these screening studies as previously mentioned in the article.

# Powder X-Ray Diffraction (PXRD)

## Temperature Variation Results


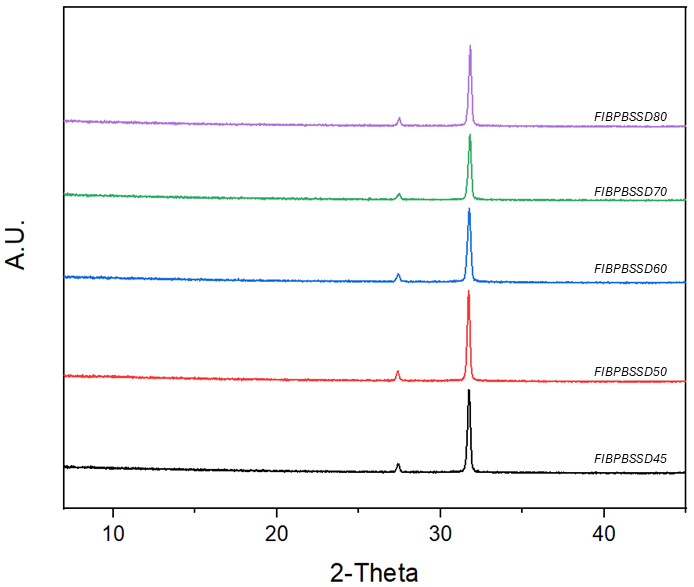


***Figure S4****, Diffractogram of spray dried fibrinogen (0.2 w/v %) in PBS buffer at varying temperatures.*

## Concentration Variation Results

**
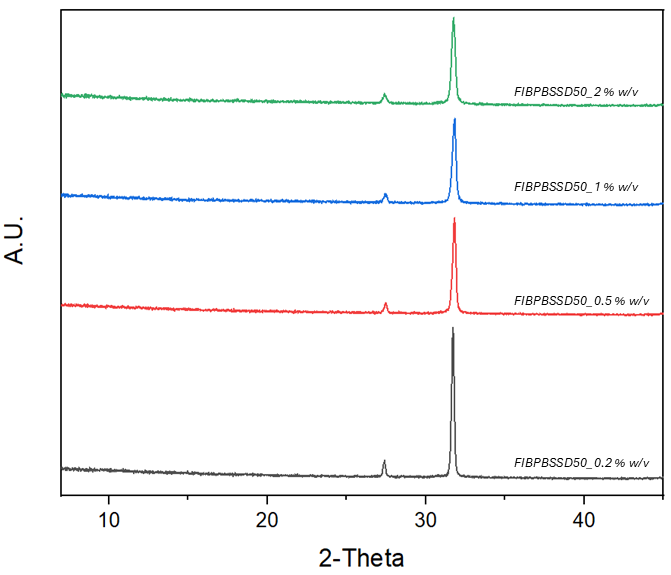
**

*Figure S5, Diffractogram of spray dried fibrinogen (T_out_ 50 °C) in PBS buffer at varying concentrations.*

# Fluorescence spectrometry

## Stability Oven: Spray dried fibrinogen spectra

**
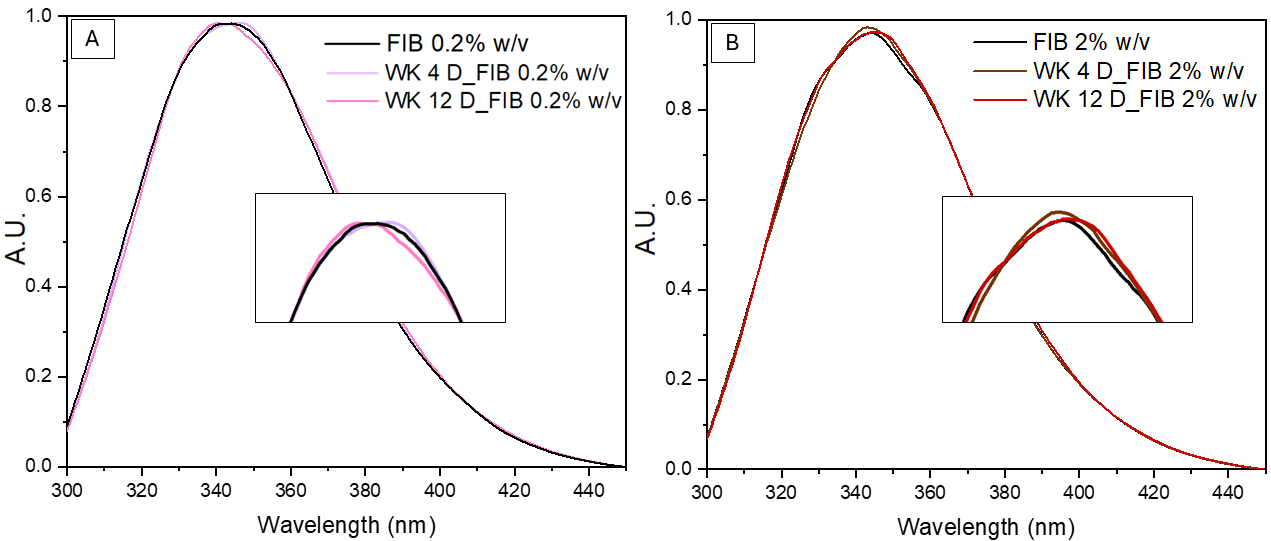
**

***Figure S6,*** *Fluorescence spectra of the stability of spray dried fibrinogen over time at different concentrations. (A) fibrinogen at 0.2 % w/v stability and (B) fibrinogen at 2 % w/v stability, Day 0, Day 30 and Day 90 in the desiccator.*

## Desiccator: Spray dried fibrinogen spectra


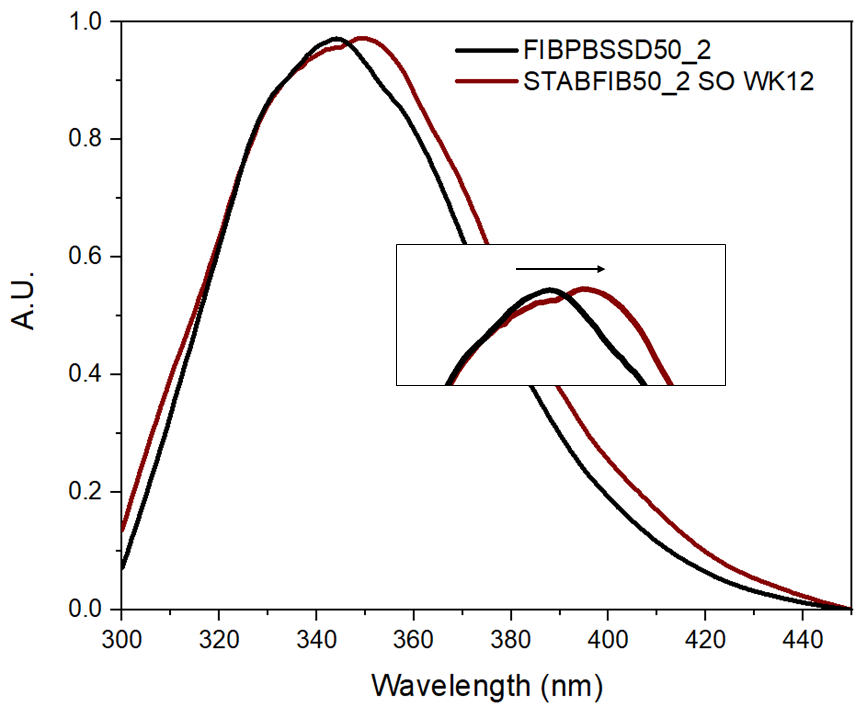


***Figure S7****, Fluorescence spectra of the stability of spray dried fibrinogen over time at different concentrations. Fibrinogen at 2 % w/v stability, Day 0, and Day 90 in the stability oven.*

*due to lack of powder for the stability oven samples only a direct comparison between Day 0 and Day 90 is illustrated above.

# Scanning Electron Microscope (SEM)


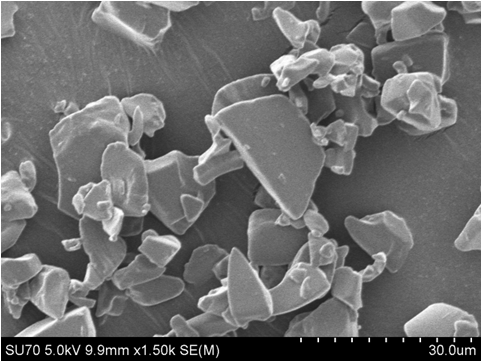


***Figure S8,*** *SEM image of fibrinogen as received.*

# Spray Drying Fibrinogen in Water

Results compare spray dried fibrinogen in PBS at 50 °C as fibrinogen had no structural alterations and the moisture content of the final powder was 2.4%. Fluorescence spectroscopy data, a shift in the maximum peak was seen along with a shift in the UV-Vis spectra baseline without the presence of PBS buffer salts. In Figure S7 (C) the FTIR data when compared to fibrinogen as received a shift in the amide II bands (1510–1580 cm^-1^) are observed, this shows changes occurring in the N-H bends of the protein affecting the integrity of the fibrinogens secondary structure spray dried solely in water. Figure S7 (D) and (E) show SEM images of fibrinogen spray dried and (F) shows both powders were fully amorphous. These results indicate that the protein was not stable spray dried in water.

***
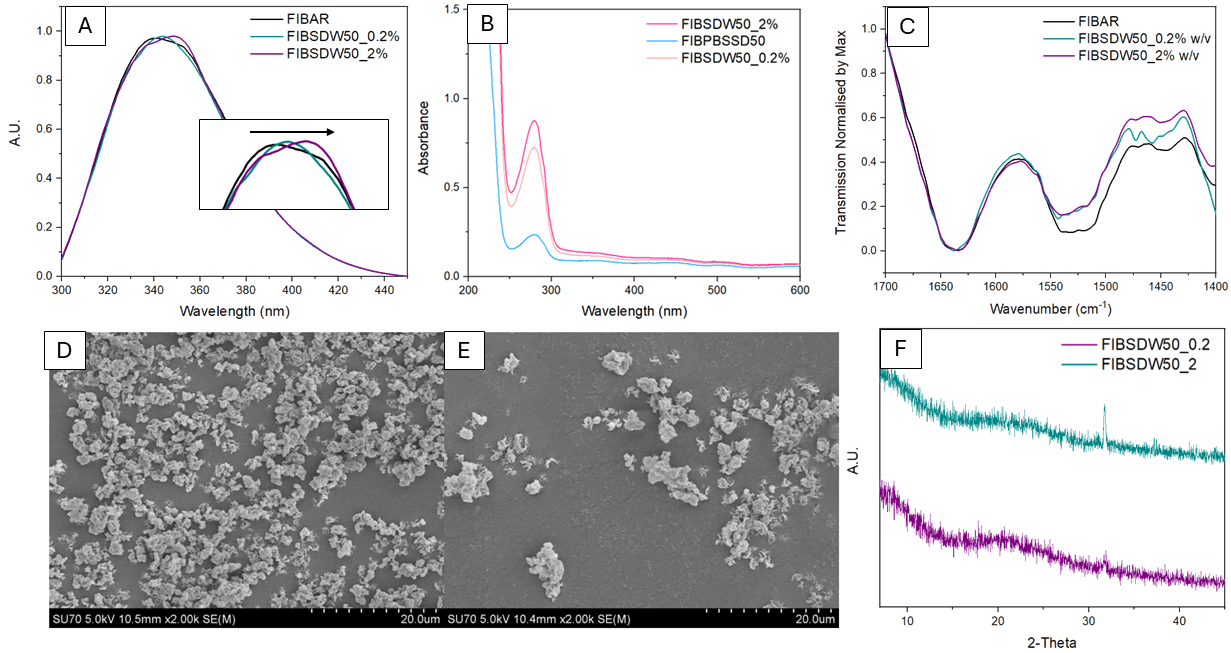
***

***Figure S9,*** *Results of spray dried fibrinogen in water at 0.2% w/v and 2% w/v. (A) Fluorescence spectroscopy plot, (B) UV-Vis spectrum comparison, (C) ATR-FTIR spectrum comparison (D) SEM image of spray dried fibrinogen at 0.2% w/v and (E) 2% w/v and (F) PXRD diffractograms of spray dried fibrinogen at 0.2% w/v and 2% w/v.*

**References:**

1. Kenmi Miyano, K.T., Hideo Nishimaki, Yoshiro Iga, *Process for heat treating fibrinogen*. 1992, GREEN CROSS Corp, Mitsubishi Tanabe Pharma Corp: United States.
